# Supplementary material for: Bacterial and Microeukaryotic Community Compositions and Their Assembly Processes in Lakes on the Eastern Qinghai-Tibet Plateau
Source: Microorganisms. 2023 Dec 23;12(1):32. doi: 10.3390/microorganisms12010032 (PMC10821157; doi:10.3390/microorganisms12010032)
Supplement: Supplementary file 1 [file microorganisms-12-00032-s001.zip › microorganisms-2728474-Supplement material.pdf]

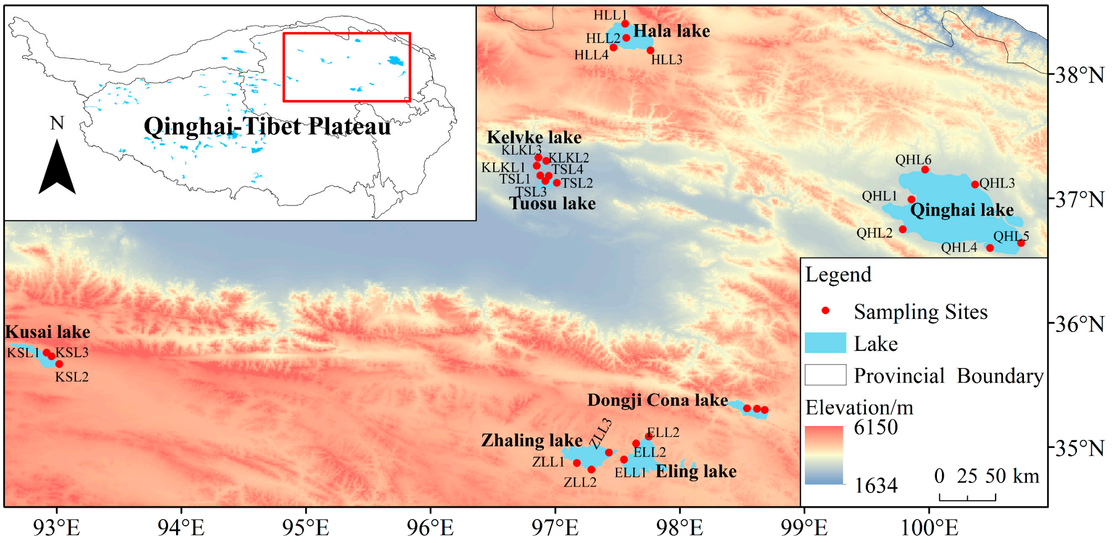

Figure S1. Map of the distributions of sampling sites across the QTP.

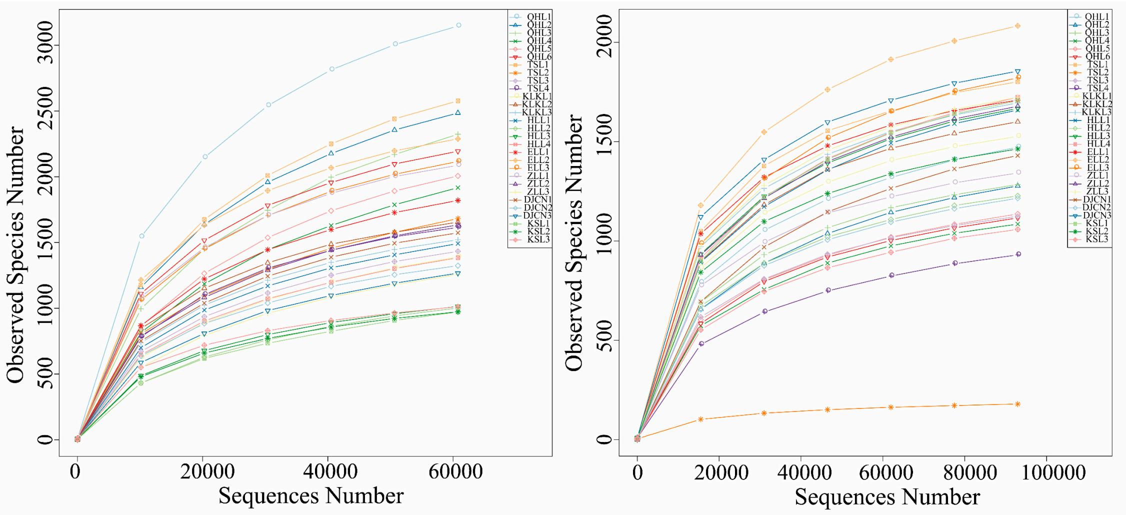

Figure S2. The rarefaction curves of the bacteria (left panel) and microeukaryotes (right panel).

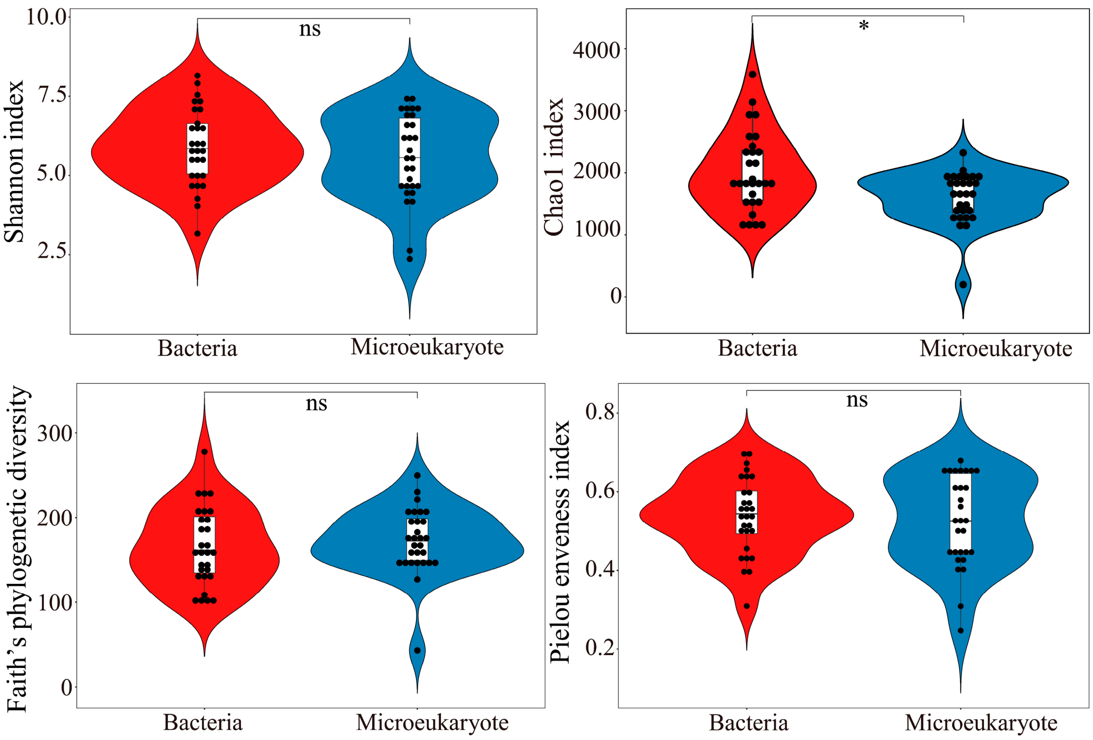

Figure S3. Comparison of the differences in four alpha diversity indices between bacterial and microeukaryotic communities using the Mann-Whitney U test. Asterisk symbols indicates significant differences between bacteria and microeukaryotes; ns represents no significant differences in alpha diversity indices between bacterial and microeukaryotic communities.

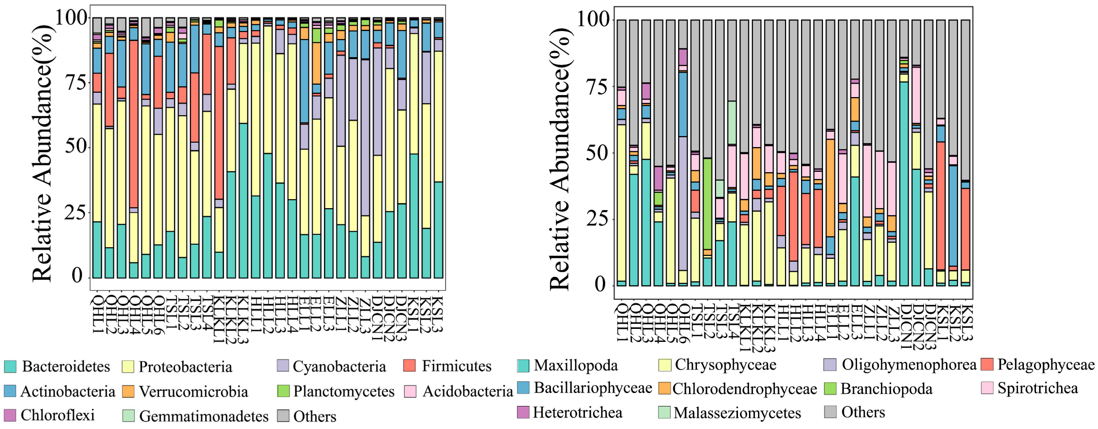

Figure S4. Top 10 taxonomic compositions of bacterial communities at the phylum level (left panel) and microeukaryotic communities at the class level (right panel).

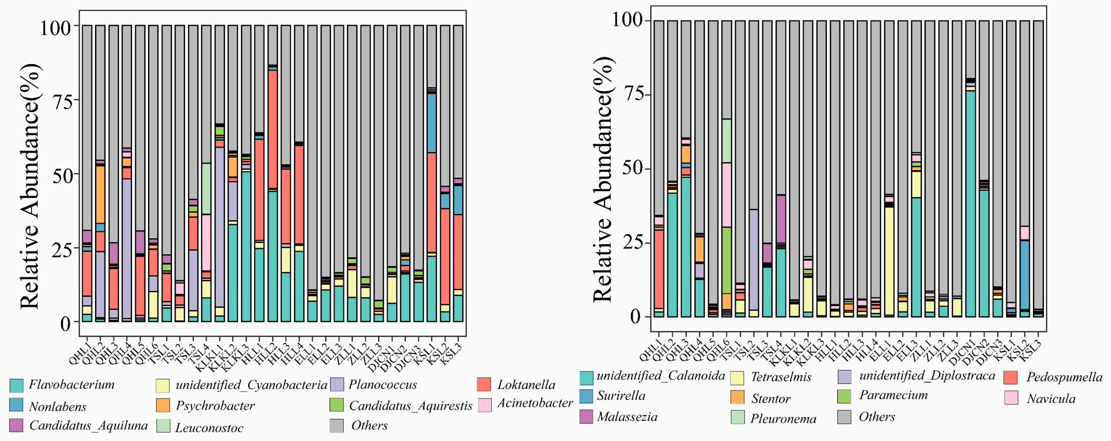

Figure S5. Top 10 taxonomic compositions of bacterial (left panel) and microeukaryotic communities (right panel) at the genus level.

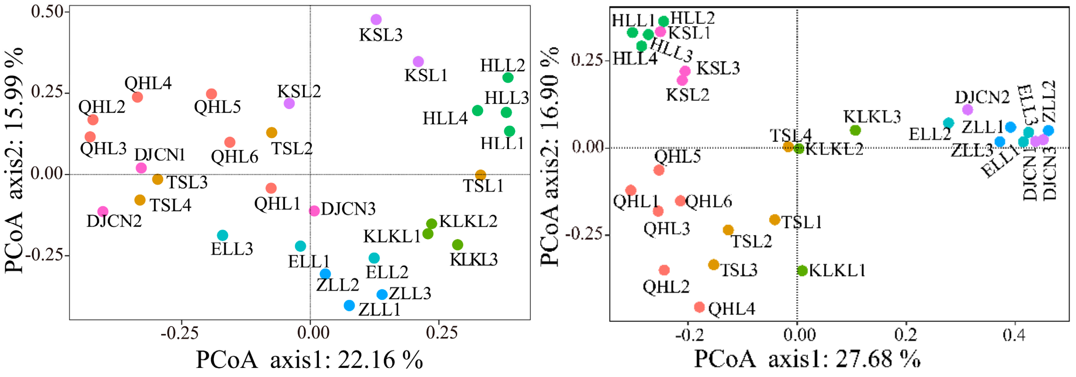

Figure S6. Principal coordinate analysis (PCoA) showed the composition differences (Bray-Curtis) of bacteria (left panel) and microeukaryotes (right panel) at the OTU level.

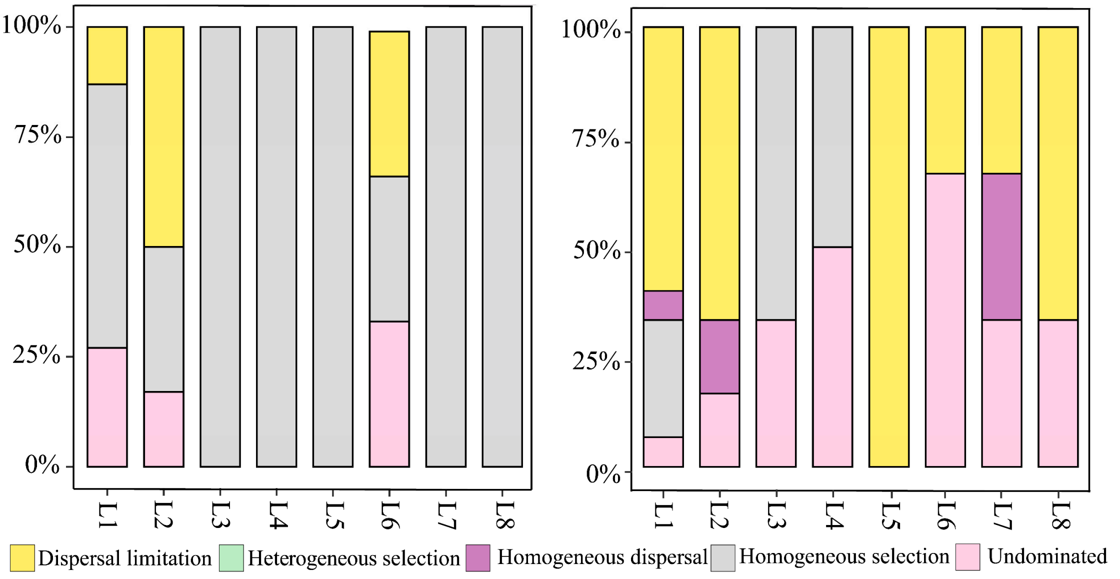

Figure S7. The contribution of the ecological processes in determining bacterial (left panel) and microeukaryotic (right panel) community assembly in each investigated lake.

Table S1 Geographic position and content of physicochemical parameters in sampled lakes..

Parameters (unit)	L1 Ranges Mean ± S.D.	L2 Ranges Mean ± S.D.	L3 Ranges Mean ± S.D.	L4 Ranges Mean ± S.D.	L5 Ranges Mean ± S.D.	L6 Ranges Mean ± S.D.	L7 Ranges Mean ± S.D.	L8 Ranges Mean ± S.D.	*P*
E/N (°)	100.16/36.91	96.90/37.14	96.85/37.29	97.59/38.27	97.65/35.00	97.29/34.88	98.61/35.03	92.98/35.72	
Temp	7.47-18.17	9.53-13.11	8.10-10.61	5.48-18.14	12.05-15.28	7.33-9.42	8.69-10.31	1.78-6.59	0.060
(℃)	11.18 ± 3.88	12.15 ± 1.51	9.07 ± 1.10	10.19± 4.68	13.66± 1.32	8.65 ± 0.94	9.27 ± 0.74	4.57 ± 2.04	
DO	2.70-7.70	5.07-7.15	5.36-7.60	6.32-8.56	5.84-6.84	6.73-7.08	8.35-10.04	6.61-7.10	0.176
(mg/ L)	6.17 ± 1.92	6.22 ± 0.75	6.79 ± 1.01	7.35 ± 0.92	6.46 ± 0.44	6.86 ± 0.16	9.41 ± 0.75	6.89 ± 0.20	
EC	5.85-14.40	18.54-75.10	0.91-19.11	0.23-3.26	0.45-0.57	0.66-0.70	0.40-0.44	6.74-11.05	**0.001**
(mS/cm)	11.75 ± 2.75	34.21±23.64	6.99 ± 8.57	2.20 ± 1.17	0.49 ± 0.05	0.68 ± 0.02	0.43 ± 0.02	8.23 ± 2.00	
TDS	5713-11689	17146-63275	746-17126	200-3349	388-454	617-691	367-428	7672-11490	**0.001**
(mg/ L)	9516 ± 2359	29204±19675	6209 ± 7719	1933± 1124	419 ± 26	653 ± 30	402 ± 25	9027 ± 1743	
SAL	4.90-10.78	16.10-69.89	0.57-16.11	0.15-2.79	0.29-0.34	0.47-0.53	0.28-0.32	6.62-7.39	**0.002**
(g/L)	9.36 ± 2.03	30.12 ± 22.96	5.75 ± 7.32	1.58 ± 0.94	0.31 ± 0.02	0.50 ± 0.02	0.30 ± 0.01	7.93 ± 1.70	
pH	9.62-9.76	8.98-9.62	8.87-9.65	9.15-10.10	9.15-9.28	9.31-9.34	9.21-9.41	9.73-9.85	**0.018**
	9.71 ± 0.06	9.44 ± 0.27	9.24 ± 0.32	9.71 ± 0.35	9.20 ± 0.06	9.23 ± 0.01	9.33 ± 0.09	9.81 ± 0.07	
ORP	2.4-814.0	2.2-384.9	27.6-93.1	70.7-127.5	101.1-154.8	131.7-148.5	97.1-127.5	73.6-83.9	0.277
(mV)	323.6±300.9	215.4 ± 152.1	52.2 ± 29.1	98.1 ± 20.4	127.5± 22.0	137.7 ± 7.6	116.4 ± 13.7	80.0 ± 4.6	
Turb	23.3-115.2	59.5-99.6	12.1-408.3	1.8-51.4	44.8-117.4	13.8-18.3	2.5-6.0	2.1-3.3	**0.006**
(NTU)	75.8 ± 36.8	80.8 ± 17.2	147.8±184.3	15.6 ± 20.8	89.8 ± 32.1	15.9 ± 1.8	4.7 ± 1.6	2.5 ± 0.6	
Chl-a	0.48-68.17	0.41-5.21	1.14-18.86	0.56-1.10	0.57-2.96	0.81-1.14	0.52-0.83	0.70-0.89	0.172
(μg/ L)	20.52± 23.80	1.80 ± 1.97	7.44 ± 8.09	0.89 ± 0.21	1.56 ± 1.01	0.99 ± 0.14	0.67±0.13	0.78 ± 0.08	
DOC	3.64-8.63	9.72-22.66	2.93-3.14	0.85-4.13	4.23-4.77	3.96-4.36	1.31-2.17	1.72-5.06	**0.002**
(mg/ L)	7.13 ± 1.77	17.78 ± 5.23	3.05 ± 0.09	2.62 ± 1.46	4.48 ± 0.22	4.12 ± 0.17	1.70 ± 0.35	3.40 ± 1.36	

TP	0.02-0.05	0.01-0.01	0.02-0.02	0.01-0.02	0.02-0.03	0.01-0.01	0.01-0.02	0.01-0.01	**0.003**
(mg/ L)	0.03 ± 0.01	0.01 ± 0	0.02 ± 0	0.02± 0.004	0.03± 0.004	0.01 ± 0	0.02 ± 0.004	0.01 ± 0	
TN	0.98-4.60	1.59-2.50	0.56-1.09	0.62-1.29	0.89-0.96	0.93-1.04	0.67-0.76	0.72-1.08	**0.007**
(mg/L)	2.05 ± 1.20	2.16 ± 0.35	0.83 ± 0.21	0.83 ± 0.27	0.92 ± 0.03	0.98 ± 0.04	0.72 ± 0.04	0.94 ± 0.16	
NH_4^+-N	0.17-0.39	0.08-0.11	0.20-0.26	0.06-0.21	0.06-0.09	0.09-0.11	0.05-0.06	0.05-0.06	**0.001**
(mg/ L)	0.30 ± 0.08	0.09 ± 0.01	0.23 ± 0.03	0.13 ± 0.06	0.08 ± 0.01	0.10 ± 0.01	0.05 ± 0.01	0.05-0.01	
NO_2^--N	0.001-0.016	0.002-0.005	0.001-0.003	0.002-0.004	0.001-0.002	0.001-0.002	0.002-0.003	0.001-0.001	0.092
(mg/L)	0.004 ± 0	0.004 ± 0	0.002 ± 0	0.003 ± 0	0.001 ± 0	0.001 ± 0	0.002 ± 0	0.001 ± 0	
NO_3^--N	0.10-1.17	0.35-0.67	0.22-0.27	0.02-0.10	0.08-0.17	0.004-0.040	0.14-0.17	0.08-0.20	**0.003**
(mg/L)	0.34±0.38	0.52±0.12	0.25±0.02	0.07± .03	0.11±0.04	0.03±0.02	0.16±0.01	0.14±0.05	

Temp, TDS, EC, ORP, Turb, DO, DOC, SAL, Chl-a, TP, TN, NH4+-N, NO3--N, and NO2--N are represented temperature, total dissolved solids, electrical conductivity, oxidation reduction potential, and dissolved oxygen, dissolved organic carbon, salinity, chlorophyll-a, total phosphorus, total nitrogen, ammonia nitrogen, nitrate-nitrogen, and nitrite nitrogen, respectively. The mS.cm-1 and the NTU are the abbreviations of MiliSiemens.cm-1 and nephelometric turbidity unit. N and E are represented the latitude and longitude respectively. Eight lakes, labeled with the prefix L- and progressive numbering, are represented Qinghai Lake, Tuosu Lake, Kelvke Lake, Hala Lake, Eling Lake, Zhaling Lake, Dongji Cona Lake, and Kusai Lake, respectively. Bold data indicate the significant difference among the sampled lakes. The Kruskal-Wallis test was used to examine differences in the environmental factors among the sampled lakes. The data denote the mean ± standard error of the mean.

Table S2 Alpha-diversity indices for bacteria and microeukaryotes in the sampled lakes.

	Simpson index		Shannon index		Chao1 index		Phylogenetic diversity		Pielou's evenness index	
	B	E	B	E	B	E	B	E	B	E
L1	0.92	0.85	6.50	4.76	2882.90	1397.61	217.42	152.58	0.58	0.46
L2	0.95	0.89	6.56	5.25	2171.68	1114.65	176.72	132.48	0.61	0.53
L3	0.82	0.97	5.24	6.93	1851.26	1833.77	153.22	176.73	0.49	0.65
L4	0.82	0.86	4.45	5.25	1502.75	1768.53	121.20	175.65	0.43	0.49
L5	0.98	0.92	7.37	6.48	2340.34	2055.44	205.12	220.81	0.67	0.60
L6	0.91	0.98	6.08	7.02	1907.01	1747.58	166.38	183.14	0.57	0.66
L7	0.92	0.73	5.77	4.91	1620.88	1695.39	139.55	183.94	0.55	0.46
L8	0.88	0.85	4.84	5.14	1211.78	1586.88	102.46	190.16	0.49	0.49
P	**0.021**	0.068	**0.021**	0.077	**0.004**	0.080	**0.003**	0.118	**0.029**	0.07

B, Bacterial; E, Microeukaryotic. Bold data indicate the significant differences among the sampled lakes using the Kruskal-Wallis test. L1 to L8 represent Qinghai Lake, Tuosu Lake, Kelvke Lake, Hala Lake, Eling Lake, Zhaling Lake, Donggi Cona Lake, and Kusai Lake, respectively.

Table S3. Differences between any two sampled lakes of bacterial and microeukaryotic communities were evaluated using the ANOSIM test.

Pairwise lakes	Bacterial communities		Microeukaryotic communities	
	r	*P*	r	*P*
L1-L2	0.35	**0.029**	0.42	**0.015**
L1-L3	0.73	**0.016**	0.88	**0.016**
L1-L4	0.75	**0.004**	0.96	**0.005**
L1-L5	1.00	**0.010**	0.62	**0.018**
L1-L6	1.00	**0.016**	0.86	**0.002**
L1-L7	1.00	**0.017**	0.37	**0.041**
L1-L8	0.59	**0.017**	0.72	**0.017**
L2-L4	0.55	**0.026**	0.05	**0.038**
L3-L4	0.80	**0.024**	1.00	**0.036**
L4-L5	1.00	**0.026**	1.00	**0.031**
L4-L6	1.00	**0.025**	1.00	**0.032**
L4-L7	1.00	**0.028**	0.94	**0.027**
L4-L8	0.70	**0.030**	0.70	**0.030**

Bold data indicate significant differences ($P < 0.05$), and any pairwise lakes have been removed with insignificant differences both in the structures of both the bacterial and micro-eukaryotic communities. Eight lakes, labeled with the prefix L- and progressive numbering are Qinghai Lake, Tuosu Lake, Kelvke Lake, Hala Lake, Eling Lake, Zhaling Lake, Donggi Cona Lake, and Kusai Lake, respectively.
